# Pharmacokinetics and Pharmacodynamics of a Depolymerized Glycosaminoglycan from *Holothuria fuscopunctata*, a Novel Anticoagulant Candidate, in Rats by Bioanalytical Methods

**DOI:** 10.3390/md19040212

**Published:** 2021-04-11

**Authors:** Shuang Liu, Taocui Zhang, Huifang Sun, Lisha Lin, Na Gao, Weili Wang, Sujuan Li, Jinhua Zhao

**Affiliations:** 1 State Key Laboratory of Phytochemistry and Plant Resources in West China, Kunming Institute of Botany, Chinese Academy of Sciences, Kunming 650201, China; liuxiaoshuang211@126.com (S.L.); zhangtaocui@mail.kib.ac.cn (T.Z.); shf112021@126.com (H.S.); linlisha@mail.kib.ac.cn (L.L.); wangweili@mail.kib.ac.cn (W.W.); lisujuan@mail.kib.ac.cn (S.L.); 2 University of Chinese Academy of Sciences, Beijing 100049, China; 3 School of Pharmaceutical Sciences, South-Central University for Nationalities, Wuhan 430074, China; gn2008.happy@163.com

**Keywords:** pharmacokinetics, pharmacodynamics, anticoagulant, fucosylated glycosaminoglycan, dHG-5, method validation

## Abstract

dHG-5 (Mw 5.3 kD) is a depolymerized glycosaminoglycan from sea cucumber *Holothuria fuscopunctata*. As a selective inhibitor of intrinsic Xase (iXase), preclinical study showed it was a promising anticoagulant candidate without obvious bleeding risk. In this work, two bioanalytical methods based on the anti-iXase and activated partial thromboplastin time (APTT) prolongation activities were established and validated to determine dHG-5 concentrations in plasma and urine samples. After single subcutaneous administration of dHG-5 at 5, 9, and 16.2 mg/kg to rats, the time to peak concentration (*T_max_*) was at about 1 h, and the peak concentration (*C_max_*) was 2.70, 6.50, and 10.11 μg/mL, respectively. The plasma elimination half-life(*T_1/2__β_*) was also about 1 h and dHG-5 could be almost completely absorbed after s.c. administration. Additionally, the pharmacodynamics of dHG-5 was positively correlated with its pharmacokinetics, as determined by rat plasma APTT and anti-iXase method, respectively. dHG-5 was mainly excreted by urine as the unchanged parent drug and about 60% was excreted within 48 h. The results suggested that dHG-5 could be almost completely absorbed after subcutaneous injection and the pharmacokinetics of dHG-5 are predictable. Studying pharmacokinetics of dHG-5 could provide valuable information for future clinical studies.

## 1. Introduction

Cardiovascular diseases are the main cause of a global health burden, with high morbidity and mortality [1,2]. Their common pathogenesis is thrombosis, the formation of obstructive thrombus, including arterial thromboembolism and venous thromboembolism (VTE) [2]. Anticoagulant is the mainstay treatment for VTE [3], which takes effect by inhibiting coagulation factor(s). Unfractionated heparin (UFH) as a traditional parenteral anticoagulant has been widely used to treat VTE for up to eight decades [4]. UFH exhibits anticoagulant activity by enhancing the effect of antithrombin (AT) in inhibiting activated coagulation factor X (FXa) and thrombin (FIIa), but its pharmacokinetics is unpredictable and clinical monitoring is required [5,6]. Compared with UFH, the pharmacokinetics of low molecular weight heparin (LMWH) is improved and clinical monitoring is not required but the risk of hemorrhagic complications remains [7,8]. The traditional oral anticoagulant warfarin also has the defects of unpredictable pharmacokinetics and high bleeding risk [9]. Although new oral anticoagulants (NOACs) have predictable pharmacokinetic characteristics, the risk of bleeding is still a main problem [10]. Taken together, bleeding risk is the common defect of these available anticoagulants, because the coagulation factor(s) inhibited is/are also essential for physiological hemostasis [11].

Great efforts have been taking to seek novel safer anticoagulants [12,13]. Researches shows that physiological hemostasis mainly depends on the extrinsic coagulation pathway, while the intrinsic coagulation pathway plays a limited role in hemostasis but can contribute to pathological thrombosis. Therefore, inhibiting the intrinsic coagulation pathway has become the main strategy for developing new generation of anticoagulants with low bleeding tendency [11]. Among others, as a selective inhibitor of intrinsic coagulation factor Xase (iXase, FIXa-FVIIIa complex), dHG-5 may be a promising anticoagulant candidate [14]. dHG-5 (Mw 5.3 kD) is a depolymerized glycosaminoglycan (HG) from sea cucumber *Holothuria fuscopunctata*. dHG-5 was prepared from natural HG by β-eliminative depolymerization and it showed potent anti-iXase activity in vitro and an antithrombotic effect in venous thrombosis model with low bleeding risk [14,15,16]. However, the pharmacokinetics of dHG-5 remains unclear. Obviously, studying its pharmacokinetic characteristics can provide further understanding of its potential value for clinical application.

The predictable pharmacokinetic profile and high bioavailability are of great importance for the druggability assessment. For small molecular drugs, the pharmacokinetics can be analyzed by chromatography, isotope labeling, and biological method, etc. However, analysis of polysaccharides has been limited due to their large molecular weight and complex structure. For instance, high performance liquid chromatography (HPLC) [17,18] is susceptible to the interference of endogenous components that have similar molecular weight as polysaccharides, and the sensitivity of ultraviolet (UV) and refractive index (RI) for polysaccharide detection is limited. As for isotope labeling [19,20], it requires special experimental conditions, and the labeled prototypes and metabolites cannot be distinguished. Whereas the biological method is based on the specific activity of the analyte in samples, e.g., LMWH, anti-FXa assay is used to study its pharmacokinetics based on its specific anti-FXa activity [21,22].

Previous studies showed that dHG-5 can selectively inhibit iXase and potently prolong the activated partial thromboplastin time (APTT) of plasma [15]. In this work, anti-iXase and APTT prolongation methods were validated for the determination of dHG-5 in plasma and urine samples, respectively. With the anti-iXase method, dHG-5 inhibits iXase, thus reducing the formation of FXa, and the generated FXa can be detected by its specific chromogenic substrate. This method has high detection sensitivity (low to 0.0625 μg/mL) for the determination of dHG-5 in bio-samples, but sophisticated pretreatment is needed for eliminating interfering substances contained in the samples. Herein, anti-iXase method was used to analyze the plasma concentration of dHG-5 at different time point postdose. Compared with anti-iXase method, APTT prolongation method is less sensitive (low to 7.5 μg/mL), but sample pretreatment is relatively simple and convenient, which was used in detection the accumulated concentration of dHG-5 in urine samples in this study. Moreover, the data on pharmacokinetics after different doses’ administration have very rarely been published [23]. We had applied anti-iXase method to rats with three different doses. Results showed that dHG-5 could be almost completely absorbed after subcutaneous injection, and it was mainly excreted in urine as the unchanged parent drug. Moreover, the pharmacokinetics and pharmacodynamics (PK/PD) of dHG-5 were well correlated after subcutaneous or intravenous administration to rats.

## 2. Results

### 2.1. Establishment and Validation of Anti-iXase Method

The anti-iXase method was established for determination of dHG-5 in rat plasma samples. The relative iXase activity-logarithmic dHG-5 plasma concentration calibration curve was of good linearity over the concentration range of 0.0625–16 μg/mL, with correlation coefficient (R^2^) > 0.99. The typical equation of standard curve was y = −14.95x + 51.49, where x and y represented the dHG-5 plasma concentration and relative iXase activity, respectively (Supplementary Figure S2 and Table S1). Accuracy and precision deviation of quality control (QC) concentrations were within ±15%, and that of the lower limit of quantification (LLOQ) (0.0625 μg/mL) did not exceed 20%.

The intra-day and inter-day precision of anti-iXase method ranged from 9.6% to 14.5% (RSD) (Table 1), and the intra-day accuracy was between 96.9% and 109.0%, as evaluated by three QC concentrations (0.131, 0.819, 12.8 μg/mL), indicating that the precision and accuracy of this method were acceptable.

To examine the effect of dilution on the concentration determination of over-ranged rat plasma samples (higher than 16 μg/mL), the diluted samples were analyzed. For the 12.5-fold diluted plasma samples, the precision (RSD%) and accuracy (%) of dHG-5 concentration were 7.3% and 88.3%, respectively. Overall, the result suggested dilution was feasible for the determination of dHG-5 in rat plasma samples.

The long-term stability of dHG-5 in rat plasma were investigated under the condition of −20 °C for 30 d. After 30 d, the recovery rate of dHG-5 was 101.4–115.4% (Table 2). It indicated that dHG-5 in rat plasma stored at −20 °C was stable for at least 30 d.

### 2.2. Pharmacokinetics of dHG-5

**Figure 1:**
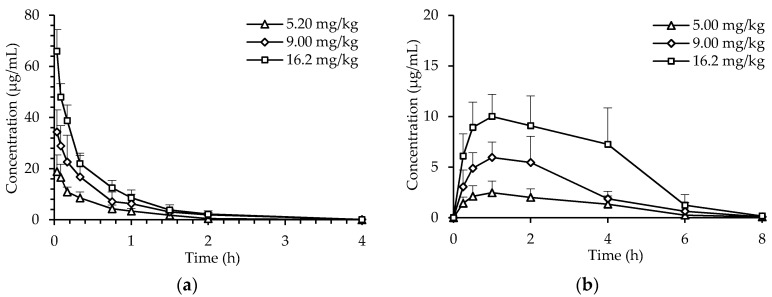
Plasma concentration-time profile of dHG-5 in rats. Rats were administrated intravenously at 5.20 mg/kg, 9.00 mg/kg, 16.2 mg/kg (**a**) and subcutaneously at 5.00 mg/kg, 9.00 mg/kg, 16.2 mg/kg (**b**). The plasma concentration of dHG-5 was calculated by anti-iXase method. Data are expressed as means ± SD (n = 5).

The pharmacokinetics of dHG-5 in rats was studied by use of the anti-iXase method. The pharmacokinetic data were best fitted with two-compartment model of in vivo metabolism. Thus, the pharmacokinetic parameters of dHG-5 were calculated according to this model. After intravenous administration (i.v.) to rats, dHG-5 plasma concentration was decreased rapidly within 20 min, and then slowly decreased to the LLOQ in 20 min-4 h post-dose (Figure 1). The elimination half-lives (*T_1/2β_*) were 0.39 ± 0.04 h (5.2 mg/kg), 0.44 ± 0.11 h (9 mg/kg), 0.48 ± 0.05 h (16.2 mg/kg) (Table 3). After subcutaneous administration (s.c.) of dHG-5, the peak plasma concentrations (*C_max_*) were reached at about 1 h (Figure 1). The *T_1/2β_* were 1.15 ± 0.34 h (5 mg/kg), 0.80 ± 0.10 h (9 mg/kg) and 1.25 ± 1.00 h (16.2 mg/kg) (Table 3). In either i.v. or s.c. administration of dHG-5, the time to peak plasma concentration (*T_max_*), central volumes of distribution (*V*_1_), and clearance (*CL*) values showed no significant difference among the three single doses. Moreover, both *C_max_* and the area under curve (AUC) of dHG-5 increased dose-dependently. Absolute bioavailability of dHG-5 subcutaneously administrated were 98.3%, 94.4%, and 104.0% at 5 mg/kg, 9 mg/kg, and 16.2 mg/kg, respectively. The reason for the phenomenon that the bioavailability was higher than 100% may be the error of the anti-iXase method itself. Compared with i.v. administration, dHG-5 given by s.c. administration was more convenient and long-lasting, suggesting s.c. administration is appropriate for clinical application in future.

### 2.3. PK/PD Correlation Analysis of dHG-5

**Figure 2:**
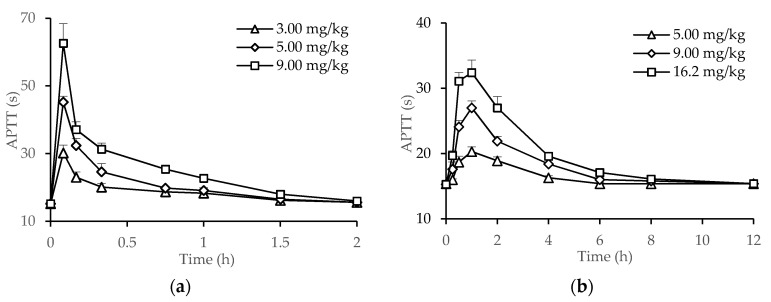
The anticoagulant activity of dHG-5 after i.v. and s.c. administration to rats. Rats were intravenously at 3.00 mg/kg, 5.00 mg/kg and 9.00 mg/kg (**a**) and subcutaneously at 5.00 mg/kg, 9.00 mg/kg and 16.2 mg/kg (**b**). Then rat plasma APTT was detected at different time points. Data were expressed as means ± SD (n = 5).

To investigate the correlation between pharmacokinetics and pharmacodynamics (PK/PD) of dHG-5, its pharmacodynamics was studied next, then the PK/PD correlation was analyzed. After i.v. and s.c. administration of dHG-5, its anticoagulant activity at different time point was evaluated by detecting the APTT of rat plasma. Rat plasma APTT values were prolonged after i.v. or s.c. administration of dHG-5 in a dose-dependent manner (Figure 2). The maximum effect occurred in about 0.083 h and 1 h after i.v. and s.c. dosing, respectively. By analyzing the PK/PD of dHG-5, rat plasma concentrations determined by the anti-iXase method were positively related with its anticoagulant activity detected by rat plasma APTT. The correlation coefficient (R^2^), a statistic representing how closely two variables co-vary, were 0.8698 (a), 0.9666 (b), 0.9044 (c), 0.7716 (d), 0.7991 (e), and 0.7863 (f) (Supplementary Figure S4).

### 2.4. Metabolite Identification

**Figure 3:**
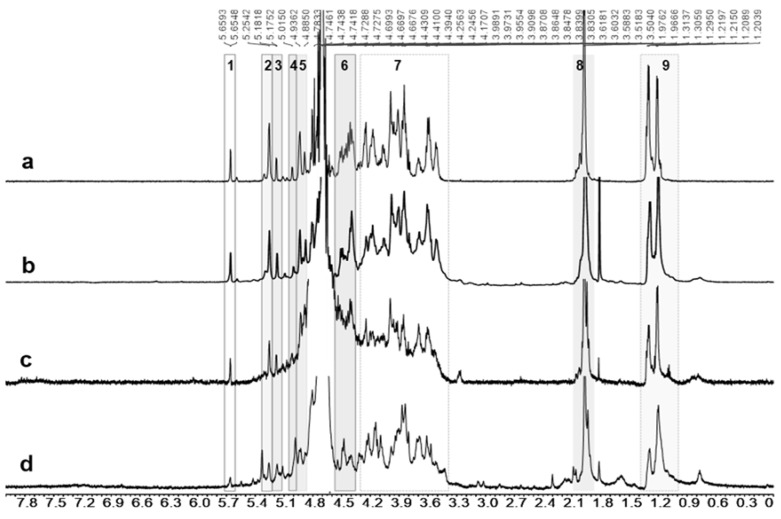
^1^H NMR spectra of dHG-5 standard and its metabolites in rat urine. It’s the dHG-5 standard for ^1^H NMR analysis was dissolved in saline (**a**) or normal rat urine (**b**), its metabolites was abstracted from urine of rats treated dHG-5 intravenously (**c**) or subcutaneously (**d**). Symbols for assignments are as follows: 1, H4 of Δ^4,5^GlcA; 2, H1 of internal Fuc_3S4S_ in saccharides chains; 3, H1 of Fuc_3S4S_ at nonreducing terminal; 4, H1 of Fuc linked to terminal alcohol; 5, H4 of internal Fuc; 6, H1 of internal GalNAc and GlcA; 7, other protons on sugar ring; 8, methyl protons in acetyl; 9, H6 of Fuc (Figure S1).

The urine (0–48 h) of rats treated with dHG-5 subcutaneously or intravenously was collected, and dHG-5 or its metabolite in urine was studied by HPLC combined with NMR analysis. The results showed that the metabolite in urine had the characteristic structures of dHG-5 (Figure 3). Comparing Figure 3c,d with Figure 3b, although ^1^H NMR signals from metabolites was not as strong as standard due to the lower amount, all characteristic protons signals can be found (Symbols “1”to “9” in Figure 3) [14]. This indicated that dHG-5 may be excreted in urine as the unchanged parent drug after s.c. and i.v. administration.

### 2.5. Establishment and Validation of APTT Prolongation Method

The APTT prolongation method was established for determination of dHG-5 in rat urine samples. The ΔAPTT-dHG-5 urine concentration calibration curves of dHG-5 were linearly fitted, over the range of 7.5–240 μg/mL. The typical standard curve was y = 0.3055 x + 0.0195 (R^2^ > 0.99), where x is the dHG-5 urine concentration and y is the prolongation of APTT (ΔAPTT) value, respectively (Supplementary Figure S3 and Table S2). Accuracy and precision of QC concentrations were within ±15%, and that of the LLOQ concentration (7.5 μg/mL) were within ±20%.

For method validation, the intra-day precision ranged from 4.4% to 13.1% (RSD) while the inter-day precision was 10.5%–11.8% (RSD) (Table 4). Moreover, the intra-day accuracy was from 110% to 115%, with deviation within 15%. The results indicated that both intra-day and inter-day precision and accuracy were acceptable.

For the stability evaluation of dHG-5 in urine, the rat urine samples were stored at room temperature for 1 d (short-term), 3 d (medium-term), and at −20 °C for 7 d (long-term). The results showed that dHG-5 in rat urine was of good stability when kept at room temperature for 1 d and at −20 °C for 7 d, while it was not stable at room temperature for 3 days (Table 5). Thus, it was feasible to store the rat urine samples at room temperature for 1 d or at −20 °C for 7 d for the APTT prolongation assay.

### 2.6. Excretion of dHG-5

**Figure 4:**
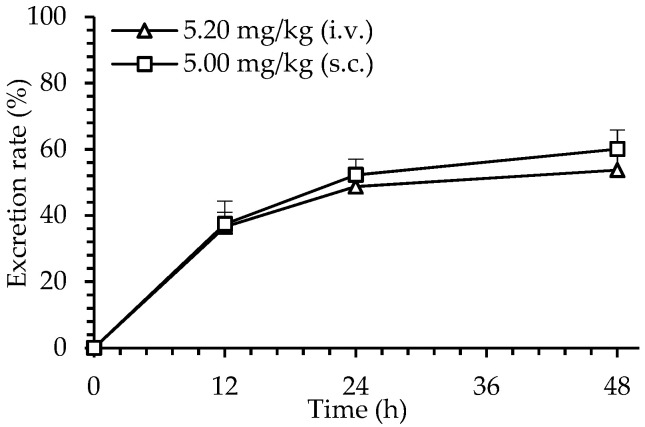
The urine excretion rate of dHG-5 in rats within 48 h. A single dose of dHG-5 was administrated subcutaneously or intravenously. Data are expressed as means ± SD (n = 5).

The excreted dHG-5 in urine was determined by APTT prolongation method. After s.c. administration of dHG-5 (5 mg/kg) to rats, the dHG-5 cumulative excretion in rat urine within 0–12, 0–24, and 0–48 h was 438.8 ± 26.0 µg, 613.7 ± 49.25 μg, and 705.3 ± 61.0 µg, respectively. In contrast, after i.v. administration of dHG-5 at 5.2 mg/kg, the cumulative excretion of dHG-5 in rat urine was 505.3 ± 115.2 µg (0–12 h), 673.6 ± 72.7 μg (0–24 h), and 742.5 ± 82.1 µg (0–48 h). The urine excretion rates of dHG-5 within 48 h were calculated to be 53.7% and 60.1% for s.c. and i.v. administration, respectively (Figure 4).

## 3. Discussion

In order to study pharmacokinetics of dHG-5, two bioanalytical methods were established and validated to determine the drug concentrations of dHG-5 in biological samples. The selectivity of the biological method is based on the specific pharmacological effect of the analyte. In this work, it is based on the anti-iXase and APTT prolongation activities of dHG-5. Previous study showed that dHG-5 had strong anti-iXase activity with the IC_50_ of 14.0 nM in vitro assay [14], which can theoretically be used for the microanalysis of dHG-5 in bio-samples. Therefore, the anti-iXase method was established and validated for determination of dHG-5 in plasma samples. Protein components contained in plasma samples have a great impact on the detection of anti-iXase activity, therefore enzyme digestion was applied to hydrolyze protein in sample pretreatment. After pretreatment, anti-iXase activity was detected, and concentrations of dHG-5 in plasma sample were calculated according to the calibration curve.

The pharmacokinetics of dHG-5 was best fitted by the two-compartment metabolic model, thus the parameters were calculated accordingly. After i.v. or s.c. administration of dHG-5 to rats, the *T_max_* and *T_1/2_* was not obviously correlated with the doses, suggesting that the elimination of dHG-5 belongs to a first-order linear kinetics. While both the *C_max_* and AUC of dHG-5 increased dose-dependently. Rat plasma APTT values were also dose-dependently prolonged after i.v. and s.c. administration of dHG-5. Absolute bioavailability of dHG-5 administrated subcutaneously were 98.3%, 94.4%, and 104.0% at a single dose of 5 mg/kg, 9 mg/kg, and 16.2 mg/kg, respectively. The reason for the phenomenon that the bioavailability was higher than 100% may be the error of the anti-iXase method itself. dHG-5 could be almost completely absorbed after s.c. administration. And it is more long-lasting compared with i.v. administration. Also, one drawback of i.v. administration was that *C_max_* was likely to be higher, thereby leading to higher APTT compared with the same dose of s.c. administration. This phenomenon after i.v. administration may increase the risk of bleeding. Therefore, s.c. administration will be more appropriate for clinical application in future. Additionally, the positive correlation between plasma concentration of dHG-5 and its anticoagulant activity indicated that the pharmacokinetics and pharmacodynamics of dHG-5 is predictable. According to the positive correlation of PK/PD, the monitoring of rats after s.c. administration may not be necessary. The results in rats provided helpful information for the routine monitoring, which will be likely not required in its future clinical application.

After i.v. or s.c. administration, the metabolite of dHG-5 was purified from accumulated rat urine samples. By HPLC and NMR analysis, it showed that dHG-5 was mainly excreted by urine as the unchanged parent drug, consisting with the report that mammal drug-metabolizing enzymes such as hyaluronidase cannot degraded the fucosylated glycosaminoglycan with branches [24]. Similarly, Toshio Imanari in 1999 reported that the structure of partially depolymerized holothurian glycosaminoglycan (DHG, Mw 12 kDa) in urine was unchanged after i.v. administration [25].

Since dHG-5 was excreted by urine as the unchanged parent drug, its excretion process can be analyzed by biological analysis. The concentrations of dHG-5 in accumulative urine sample were determined by the APTT prolongation method, which had been established and validated. We noticed that the urine stability results (Table 5) showed very high recovery rates when stored at room temperature for 3 d. This phenomenon might be attributed to the presence of bacteria and inorganic salts in the urine, which affect the turbidity during plasma coagulation. The result showed that dHG-5 could be mainly excreted within 48 h (about 60%).

Compared with the anti-Xa assay by amidolytic assay [26], there were the similarities and differences. Firstly, the similarities. Both of the two methods were of bioanalytical methods, which could be used for determination of the analyte’s concentrations in plasma. And the analytes were marine-derived polysaccharides. Next, the differences. The anti-iXase method was based on anti-iXase activity of dHG-5 while the anti-Xa assay by amidolytic assay was based on the anti-Xa activity of fucoidan. What is more, the components in blood plasma do not affect the spectra of the reaction products of the heparin kit in the anti-Xa assay by amidolytic assay, while the protein components contained in plasma samples have a great impact on the detection of anti-iXase activity. Compared with other anticoagulants already used in clinical practice, the main advantages of dHG-5 were not only in its new anticoagulant target iXase, but also in the lower bleeding tendency [14]. The anti-FXa assay is commonly used to study the pharmacokinetics based on its specific anti-FXa activity of LMWH in normal volunteers [21]. The pharmacokinetic stduy of dHG-5 by anti-iXase method in this work was based on the anticoagulant target iXase, while the anti-FXa assay studied the pharmacokinetics of LMWH by acting on FXa. Both the anti-iXase method and anti-FXa assay are bioanalytical methods. Nevertheless, the anti-iXase method was more challenging than the anti-FXa assay due to iXase being more complicated than Xa. Thus, the anti-iXase method may provide effective information for future clinical pharmacokinetics of dHG-5.

## 4. Materials and Methods

### 4.1. Chemicals and Reagents

nHG was extracted and purified from sea cucumber *Holothuria fuscopunctata* as previously described [27]. dHG-5 (Mw 5.3 kD) was one of the low molecular weight depolymerized products of nHG (dHGs), which was prepared by the β-eliminative depolymerization method [16]. It was mainly composed of the oligosaccharides: oHG-5, oHG-8, oHG-11, oHG-14, oHG-17, oHG-20, oHG-23, oHG-26, and oHG-29 with the general structure *L*-Fuc_3S4S_-α1,3-L-Δ^4,5^GlcA-α1,{3-*D*-GalNAc_4S6S_-β1,4-[*L*-Fuc_3S4S_-α1,]3-*D*-GlcA-β1,}_n_-3-*D*-GalNAc_4S6S_-β1,4-[*L*-Fuc_3S4S_-α1,] 3-*D*-GlcA-ol (n = 0–8) [14,15] (Supplementary Figure S1). Biophen FVIII:C kit was purchased from Hyphen Biomed (Neuville Sur Oise, France). Recombinant coagulation factor VIII was from Bayer HealthCare LLC (Berkeley, CA, USA). The APTT kits and human coagulation control plasma were from Teco Medical (Munich, Germany). Calcium chloride dihydrate was from Sigma (St. Louis, IL, USA). Alkaline protease was produced by Solarbio (Beijing, China). Trisodium citrate dihydrate and NaOH were from Damao (Tianjin, China) Amberlite FPA98 ion exchange resin was produced by Rohm and Haas Company (Philadelphia, PA, USA). Sephadex G-25 resin was from GE Healthcare Life Sciences (Uppsala, Sweden). All chemical reagents were of analytical grade.

### 4.2. Animals

Sprague-Dawley (SD) male rats, weighing 250–280 g, were purchased from Hunan SJA Laboratory Animal Co., Ltd., Changsha, China (License No. SCXK (Xiang) 2016-0002). Animals were maintained in an air-conditioned animal house at the temperature of 24 ± 2 °C, relative humidity of 40% to 60%. Animals were housed six per cage, eating and drinking ad libitum, and allowed to accommodate for about a week prior to experiment. Experiments were approved by the Animal Ethics Committee of Kunming Institute of Botany, Chinese Academy of Sciences.

### 4.3. Pharmacokinetics and Pharmacodynamics (PK/PD) of dHG-5

#### 4.3.1. Anti-IXase Method for dHG-5 Plasma Concentration Analysis

Preparation of Standard Samples: Stock solution of dHG-5 (1.92 mg/mL) was prepared with saline. The dHG-5 standard samples at 0.0625, 0.125, 0.5, 1, 8 and 16 µg/mL were prepared from stock solution which was diluted with drug-free rat plasma. Quality control (QC) samples at 0.131, 0.819, and 12.8 µg/mL were similarly prepared.

Pretreatment of Plasma Samples: The rat plasma sample (200 μL) was centrifuged at 10,000 rpm for 10 min, then the supernatant (150 µL) was collected to another tube. In order to hydrolyze the plasma proteins that would interfere the detection, 5% alkaline protease solution (16.5 µL) and 1 M NaOH solution (3 µL) were added, mixed by vortex, and the mixed solution was sealed, and incubated at 50 °C for 15 h. After this, the sample was centrifuged at 13,000 rpm for 10 min, then 90 µL of the supernatant was collected. The supernatant was mixed with 10 µL of 0.4 M CaCl_2_ solution, and the pretreated sample was ready for anti-iXase activity.

Bioanalysis: Anti-iXase activity was determined by chromogenic substrate method [27,28,29,30]. Thus, a 30 µL pretreated sample (drug-free sample as control), 30 μL 2 IU/mL FVIII and 30 µL 60 nM FIXa (containing phosphatidylcholine/phosphatidylserine, Ca^2+^, human thrombin) were added in a 96-well microtiter plate and incubated at 37 °C for 2 min. Then 30 µL 50 nM FX (containing thrombin inhibitor) was added, and incubated at 37 °C for 1 min. Then the FXa activity which presents residual iXase activity, was detected by the addition of 30 μL SXa-11 (FXa chromogenic substrate). Optical density (OD) at 405 nm of the mixed solution was recorded by a Microplate Reader (Bio-Tek ELX 808, Vermont, USA) with a kinetic method (read per 30 s for 2 min at 37 °C). The change rate in OD (ΔOD/min) of each sample was calculated, which represented the activity of iXase. ΔOD/min of the control was set as 100%, and the relative iXase activity of pretreated samples (% of control) was calculated. Finally, the concentration of dHG-5 in each sample was calculated according to the calibration curve introduced above.

Method Validation: Anti-iXase method was validated according to the standard guideline [31]. Linearity, accuracy, precision, dilution reliability, and stability were included in validation.

The calibration curve was fitted by linear regression of the relative iXase activity (% of control) and logarithmic dHG-5 plasma concentration of a series of standard samples. The lowest plasma concentration of dHG-5 in the calibration curve was considered as the lower limit of quantification (LLOQ), whose accuracy and precision deviations were required to be within 20%.

Accuracy and precision were evaluated using three QC concentrations. The accuracy was calculated as (mean measured concentration/labeled concentration) × 100%, and the precision was assessed by relative standard deviation (RSD).

The dHG-5 levels in some rat plasma samples were beyond the highest concentration of the calibration curve, thereby validation of dilution reliability was conducted. Samples (dHG-5 at 64 µg/mL) were diluted 12.5-fold, the activity of diluted samples were tested, and the concentrations were calculated from the calibration curve.

The stability of dHG-5 in rat plasma was verified by three QC concentrations at −20 °C for 30 d.

All the experiments were repeated at least three times. For the above validation experiments, both the accuracy and precision of the tested concentrations should be within ±15%, and that of the concentration around LLOQ within ±20% is acceptable.

#### 4.3.2. Pharmacokinetics of dHG-5

Rats were weighed and randomly assigned into six groups (n = 5/group). For s.c. administration, three animal groups were dosed with dHG-5 at 5 mg/kg, 9 mg/kg and 16.2 mg/kg. For i.v. injection, another three groups were dosed with dHG-5 at 5.2 mg/kg, 9 mg/kg, and 16.2 mg/kg. After single dose s.c. administration, blood (0.45 mL) was collected from the ocular vein and anticoagulated with 50 μL of 3.8% sodium citrate solution at 0, 0.25, 0.5, 1, 2, 4, 6, and 8 h. Similarly, for single dose i.v. administration, blood collection was conducted before and at 0.033, 0.083, 0.167, 0.333, 0.75, 1, 1.5, 2, 4, 6, and 8 h after dosing. Each blood sample was immediately centrifuged at 3000× *g* for 10 min to obtain the plasma, then stored at −20 °C before analysis. The plasma concentrations of dHG-5 were determined by anti-iXase method as described in 4.3.1. The selection of the most suitable compartment model was based on the minimum Akaike’s information criterion (*AIC*), and the pharmacokinetic parameters were analyzed from the concentration-time data using a two-compartment pharmacokinetic model by DAS 2.0 software (Shanghai, China). Absolute bioavailability of dHG-5 at different doses was acquired by the formula: *F*% = (AUC_s.c._ × dose_i.v._)/(AUC_i.v._ × dose_s.c._) × 100. The statistical significance of results was determined by GraphPad Prism 7.00 software using one-way analysis of variance (ANOVA) followed by Dunn’s multiple comparisons test. *p* values of less than 0.05 were considered significant, and *p* values of more than 0.05 were considered not significant.

#### 4.3.3. Pharmacodynamics of dHG-5

Rats were randomly divided into six groups (n = 5/group). Animals were grouped and treated with dHG-5 as described in 4.3.2. After i.v. (or s.c.) administration of dHG-5, blood (0.27 mL) was drawn from the ocular vein and anticoagulated with 30 µL of 3.8% sodium citrate solution at 0, 0.083, 0.167, 0.333, 0.75, 1, 1.5, 2, 4, 6, and 8 h (or 0, 0.25, 0.5, 1, 2, 4, 6, 8, 12 h). Each blood sample was then centrifuged at 1800× *g* for 15 min to obtain the plasma for APTT assay. The APTT of rat plasma samples was detected with a coagulometer (TECO MC-4000, Munich, Germany) as previously reported [15,32]. Moreover, the PK/PD correlation was analyzed using the pharmacokinetic data given in Section 4.3.2.

### 4.4. Metabolites and Excretion Analysis

#### 4.4.1. Metabolite Identification

Metabolization of dHG-5 was analyzed by HPLC and NMR method. After administration of 5.2 mg/kg (i.v.) or 5 mg/kg (s.c.) dHG-5 to rats (n = 5), the urine sample during 0–48 h was collected and concentrated. Then, the sample was purified with a FPA98 strong ion-exchange chromatography (1.5 × 15 cm), sequentially eluted with NaCl solution at 0, 0.5, 0.8, 1.0, 1.2, 1.5, and 2.0 M at 2-fold bed volume (BV). Each eluted fraction was desalted by a Sephadex G-25 column, and analyzed by HPLC with a Superdex peptide 10/300 G L column and a differential refractive index detector (RID), eluted by 0.2 M NaCl eluent at the flow rate of 0.4 mL/min. The eluates contained dHG-5 were lyophilized into powders and analyzed by NMR with a Bruker Advance 600 MHz spectrometer and a ^1^H/^13^C dual probe in FT mode at 298 K.

#### 4.4.2. APTT Prolongation Method for dHG-5 Urine Concentration Analysis

Preparation of standard samples: Mother solution of dHG-5 at 0.96 mg/mL was prepared with drug-free rat urine which was collected for 24 h from normal rats. A series of rat urine standard samples at 7.5, 15, 30, 60, 120, and 240 µg/mL were obtained by diluting the mother solution with drug-free rat urine. Likewise, rat urine QC samples (15, 45 and 180 µg/mL) were prepared.

Pretreatment of Urine Samples: The rat urine sample was centrifuged (4000 rpm, 5 min) to obtain the supernatant, then filtering the supernatant through a 0.45 µm microporous membrane, the filtrate was collected as the pretreated sample for APTT prolongation detection.

Bioanalysis: A 5 µL pretreated sample (drug-free rat urine sample as control) and 45 µL of human coagulation control plasma were pipetted into a blood clotting cup, then incubated at 37 °C for 2 min. Then, 50 µL of APTT reagent was added and incubated at 37 °C for 3 min. The clotting time was recorded after the addition of CaCl_2_ reagent (50 µL). The prolongation of APTT (ΔAPTT) of each sample was defined by subtracting the APTT of control. The calibration curve ΔAPTT-dHG-5 urine concentration was introduced above, and the dHG-5 concentrations in urine samples were calculated accordingly.

Method validation: Similar to the validation of anti-iXase method as described in 4.3.1, APTT prolongation method was validated in linearity, accuracy, precision and stability.

#### 4.4.3. Excretion Assay

Ten rats were randomly allocated to two group (n = 5/group), in which dHG-5 was administrated at 5.2 mg/kg (i.v.) or 5 mg/kg (s.c.). After dosing, the rats were housed in separate metabolic cages for urine collection, and urine during 0–12, 12–24, 24–48 h was collected. Urine was centrifuged at 4000 rpm for 5 min, and the supernatant was filtered via a 0.45-µm microporous membrane. The pretreated urine sample was then frozen at −20 °C until APTT prolongation detection.

## 5. Conclusions

In this work, two biological methods were developed, validated, and then applied to study the pharmacokinetics of dHG-5 in rats. To our best knowledge, the pharmacokinetics of dHG-5 studied by the anti-iXase method and the excretion of dHG-5 studied by the APTT prolongation method are reported for the first time. The results showed that, by s.c. administration of dHG-5 to rats, the absolute bioavailability of dHG-5 was high (above 90%), suggesting that dHG-5 was almost completely absorbed. *C_max_*, AUC, and rat plasma APTT values increased dose-dependently, indicating that the PK/PD of dHG-5 was well correlated. In addition, dHG-5 was mainly excreted by urine as the unchanged parent drug, and about 60% of it was excreted within 48 h. These results suggested that dHG-5 has predictable pharmacokinetic characteristics, and that s.c. administration should be an appropriate delivery method for its possible clinical application in the future. Together, these results provide important insights into the PK/PD of dHG-5, implying the potential druggability of dHG-5.

## Figures and Tables

**Table 1:** Precision and accuracy of anti-iXase method.

Nominal Concentration (µg/mL)	Intra-Day Accuracy (%, n = 5)	Intra-Day Precision (RSD%, n = 5)	Inter-Day Precision (RSD%, n = 15)
0.131	99.8	14.5	13.6
0.819	109.0	12.6	11.4
12.8	96.9	9.6	12.1

**Table 2:** Stability of dHG-5 in rat plasma (means ± SD, n = 3).

Nominal Concentration (μg/mL)	Recovery Rate (%) of dHG-5 after 30 d at −20 °C
0.131	101.4 ± 11.1
0.819	115.4 ± 3.8
12.8	109.4 ± 9.0

**Table 3:** Main pharmacokinetic parameters of dHG-5 after i.v. and s.c. administration (means ± SD, n = 5).

Parameters ^1^	5.2 mg/kg		5 mg/kg	9 mg/kg		16.2 mg/kg	
	i.v.		s.c.	i.v.	s.c.	i.v.	s.c.
*T_1/2α_* (h)	0.25 ± 0.20		0.94 ± 0.33	0.29 ± 0.20	0.64 ± 0.10	0.07± 0.05	1.08 ± 0.94
*T_1/2β_* (h)	0.39 ± 0.04		1.15 ± 0.34	0.44 ± 0.11	0.80 ± 0.10	0.48 ± 0.05	1.25 ± 1.00
*C_max_* (μg/mL)	19.08 ± 6.23		2.70 ± 1.22	34.40 ± 8.56	6.50 ± 2.10	65.98 ± 8.36	10.11 ± 2.22
*T_max_* (h)	0.04 ± 0.02		0.90 ± 0.22	0.03 ± 0.00 *	1.00 ± 0.61 **^#^**	0.03 ± 0.00 *	1.10 ± 0.55 **^#^**
*V*_1_(L/kg)	0.26 ± 0.09		0.82 ± 0.32	0.23 ± 0.03 *	0.56 ± 0.19 **^#^**	0.19 ± 0.04 *	0.26 ± 0.23 **^#^**
*CL* (L/h/kg)	0.52 ± 0.12		0.40 ± 0.20	0.54 ± 0.30 *	0.50 ± 0.17 **^#^**	0.49 ± 0.03 *	0.43 ± 0.21 **^#^**
AUC_0-t_ (μg/mL∙h)	9.51± 2.21		9.17 ± 1.38	19.59 ± 10.48	17.93 ± 6.15	30.88 ± 2.15	34.52 ± 9.50
AUC_0-__∞_ (μg/mL∙h)	10.53 ± 2.40		9.95 ± 1.41	21.07 ± 10.67	19.89 ± 6.64	33.23 ± 1.94	34.43 ± 10.51
*F* (%)		/	98.3	/	94.4	/	104.0

^1^*C_max_*: the peak concentration; *T_max_*: the time to peak concentration; AUC_0__–t_: area under curve from time zero to the last sampling time; AUC_0-__∞_: area under curve from time zero to infinity; *T_1/2__α_*: the plasma distribution half-life; *T_1/2__β_*: the plasma elimination half-life; *V*_1_: central volume of distribution; *CL*: clearance; *F*: bioavailability; *: *p* > 0.05 vs. 5.2 mg/kg (i.v.); ^#^ *p* > 0.05 vs. 5 mg/kg (s.c.) (One-way ANOVA, Dunn’s multiple comparisons test).

**Table 4:** Precision and accuracy of APTT prolongation method.

Nominal Concentration (µg/mL)	Intra-Day Accuracy (%, n = 5)	Intra-Day Precision (RSD%, n = 5)	Inter-Day Precision (RSD%, n = 15)
15	115.0	4.4	10.7
45	110.0	11.2	11.8
180	115.0	13.1	10.5

**Table 5:** Stability of dHG-5 in rat urine (means ± SD, n = 3).

Nominal Concentration (µg/mL)	Recovery Rate (%) of dHG-5		
	Short-Term (r.t., 1 d)	Medium-Term (r.t., 3 d)	Long-Term (−20 °C, 7 d)
15	89.5 ± 0.05	754.0 ± 0.86	112.0 ± 2.57
45	104.0 ± 0.07	560.0 ± 0.62	128.0 ± 5.73
180	123.0 ± 0.05	191.0 ± 0.26	109.0 ± 15.60

## Data Availability

All data is contained within this article and Supplementary Materials.

## Supplementary Materials

The following are available online at https://www.mdpi.com/article/10.3390/md19040212/s1, Figure S1: Structure (A), HPGPC profile (B) & ^1^H NMR spectra (C) of dHG-5 [14,15]. Figure S2: The calibration curves for dHG-5 over the concentration range of 0.0625–16 µg/mL in rat plasma. Figure S3: The calibration curves for dHG-5 over the concentration range of 7.5–240 μg/mL in rat urine. Figure S4: The relationship between dHG-5 plasma concentration and plasma APTT. dHG-5 plasma concentration was determined by the anti-iXase method, and the anticoagulant activity was detected using rat plasma, after intravenous administration at 3.00 mg/kg (a), 5.00 mg/kg (b) and 9.00 mg/kg (c) and subcutaneous administration to rats at 5.00 mg/kg (d), 9.00 mg/kg (e) and 16.2 mg/kg (f). Table S1: Regression equations for determination of dHG-5 concentration in rat plasma. Table S2: Regression equations for determination of dHG-5 concentration in rat urine.
