# Supplementary material for: Pharmacokinetics and Pharmacodynamics of a Depolymerized Glycosaminoglycan from Holothuria fuscopunctata, a Novel Anticoagulant Candidate, in Rats by Bioanalytical Methods
Source: Mar Drugs. 2021 Apr 11;19(4):212. doi: 10.3390/md19040212 (PMC8069088; doi:10.3390/md19040212)
Supplement: Supplementary file 1 [file marinedrugs-19-00212-s001.pdf]

Supporting Information

Pharmacokinetics and Pharmacodynamics of a Depolymerized Glycosaminoglycan from Holothuria Fuscopunctata, a Novel Anticoagulant Candidate, in Rats by Bioanalytical Methods

Shuang Liu [1,2,†], Taocui Zhang [1,2,†], Huifang Sun [1,2], Lisha Lin [1,2], Na Gao [3], Weili Wang [1,2], Sujuan Li [1,2] and Jinhua Zhao [3,*]

[1] State Key Laboratory of Phytochemistry and Plant Resources in West China, Kunming Institute of Botany, Chinese Academy of Sciences, Kunming 650201, China; liuxiaoshuang211@126.com (S.L.); zhangtaocui@mail.kib.ac.cn (T.Z.); shf112021@126.com (H.S.); linlisha@mail.kib.ac.cn (L.L.); wangweili@mail.kib.ac.cn (W.W.); lisujuan@mail.kib.ac.cn (S.L.)

[2] University of Chinese Academy of Sciences, Beijing 100049, China

[3] School of Pharmaceutical Sciences, South-Central University for Nationalities, Wuhan 430074, China; gn2008.happy@163.com (N.G.)

[†] These authors contributed equally to this work.

[*] Correspondence: zhao.jinhua@yahoo.com (J.Z.)

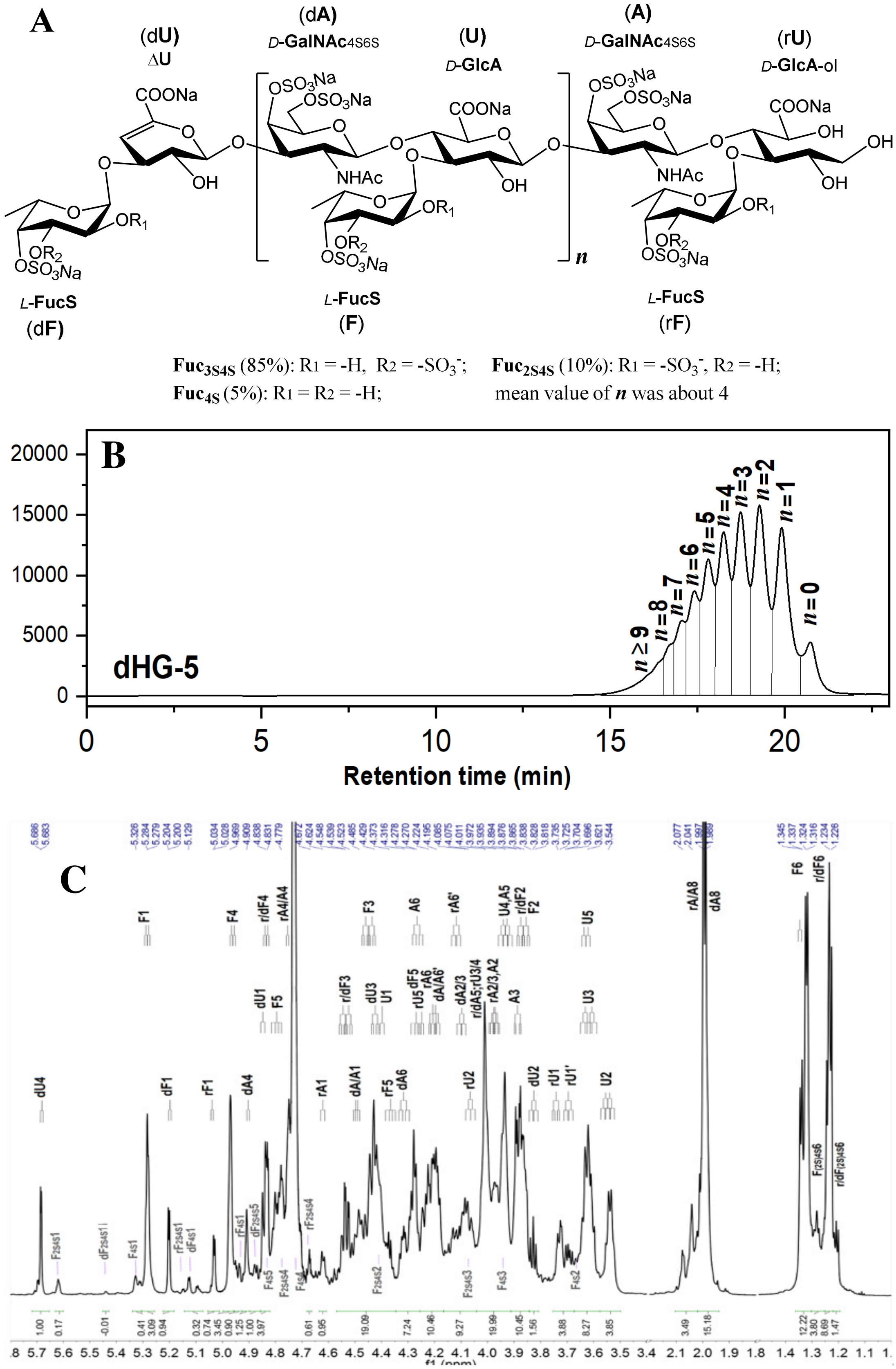

Figure S1. Structure (A), HPGPC profile (B) & 1H NMR spectra (C) of dHG-5 [14, 15].

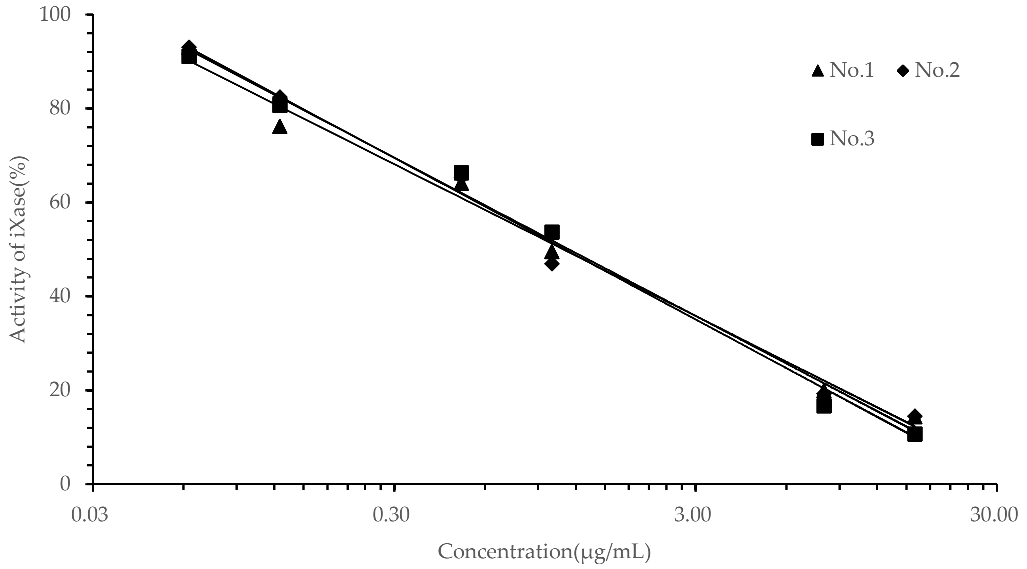

Figure S2. The calibration curves for dHG-5 over the concentration range of 0.0625-16 μg/mL in rat plasma.

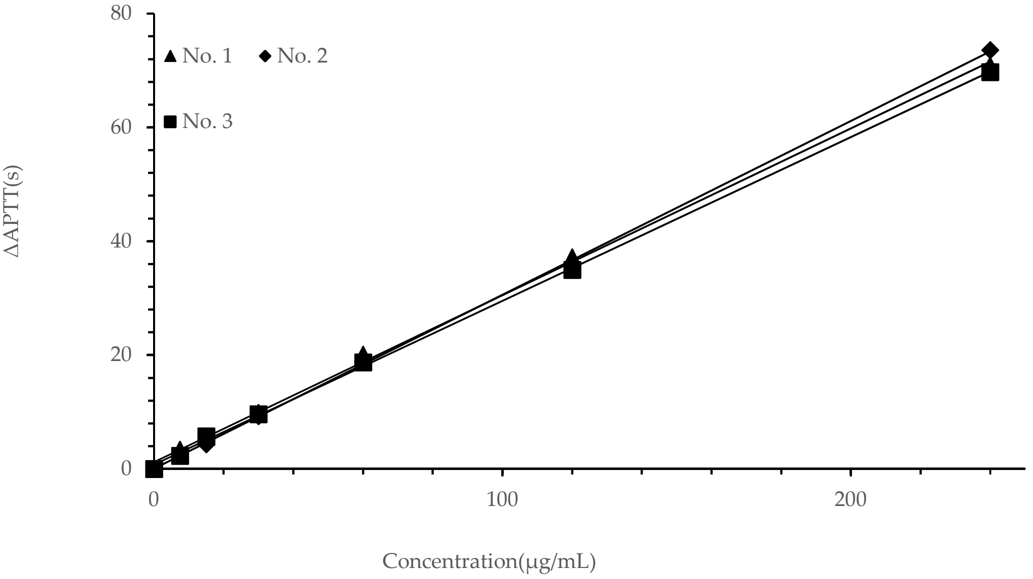

Figure S3. The calibration curves for dHG-5 over the concentration range of 7.5-240 μg/mL in rat urine.

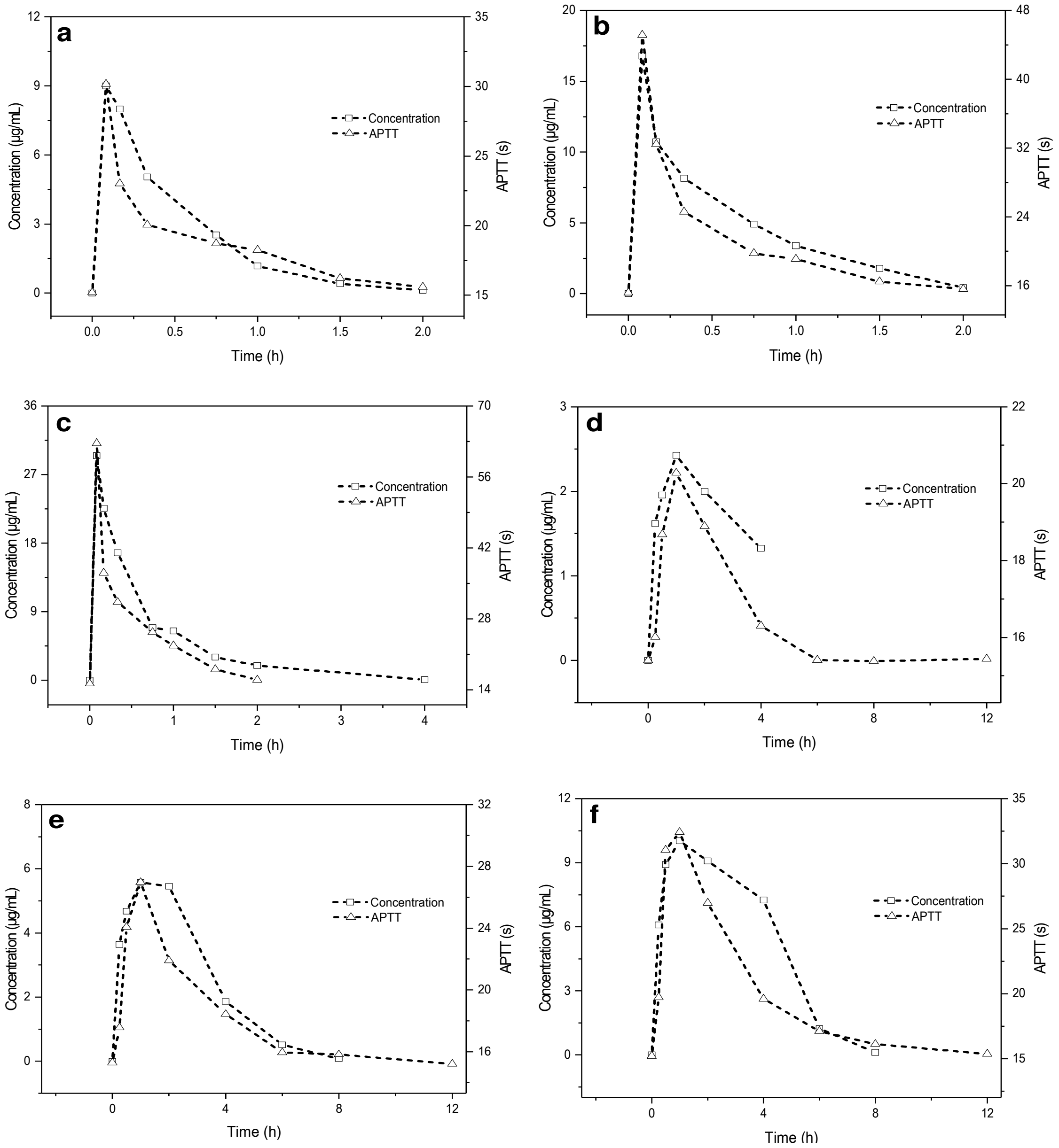

Figure S4. The relationship between dHG-5 plasma concentration and plasma APTT. dHG-5 plasma concentration was determined by the anti-iXase method, and the anticoagulant activity was detected using rat plasma, after intravenous administration at 3.00 mg/kg (**a**), 5.00 mg/kg (b) and 9.00 mg/kg (**c**) and subcutaneous administration to rats at 5.00 mg/kg (**d**), 9.00 mg/kg (e) and 16.2 mg/kg (f). Data were expressed as means ± SD (n=5).

Table S1. Regression equations for determination of dHG-5 concentration in rat plasma.

No.	Regression equation	R^2
1	$y = -14.04\ x + 51.24$	0.9902
2	$y = -14.63\ x + 51.91$	0.9904
3	$y = -14.95\ x + 51.49$	0.9916

Table S2. Regression equations for determination of dHG-5 concentration in rat urine.

No.	Regression equation	R^2
1	$y = 0.2931\ x + 1.1932$	0.9989
2	$y = 0.3055\ x + 0.0195$	0.9997
3	$y = 0.2878\ x + 0.7282$	0.9994
